# Predicting Protein-Protein Interactions Using BiGGER: Case Studies

**DOI:** 10.3390/molecules21081037

**Published:** 2016-08-09

**Authors:** Rui M. Almeida, Simone Dell’Acqua, Ludwig Krippahl, José J. G. Moura, Sofia R. Pauleta

**Affiliations:** 1 UCIBIO, REQUIMTE, Departamento de Química, Faculdade de Ciências e Tecnologia, NOVA, 2829-516 Caparica, Portugal; rui.almeida@fct.unl.pt; 2 Department of Chemistry, University of Pavia, Via Taramelli 12, 27100 Pavia, Italy; simone.dellacqua@unipv.it; 3 CENTRIA, Departamento de Informática, Faculdade de Ciências e Tecnologia, NOVA, 2829-516 Caparica, Portugal; a4338@fct.unl.pt

**Keywords:** protein-protein interactions, BiGGER, docking, electron transfer complexes, molecular recognition, NMR

## Abstract

The importance of understanding interactomes makes preeminent the study of protein interactions and protein complexes. Traditionally, protein interactions have been elucidated by experimental methods or, with lower impact, by simulation with protein docking algorithms. This article describes features and applications of the BiGGER docking algorithm, which stands at the interface of these two approaches. BiGGER is a user-friendly docking algorithm that was specifically designed to incorporate experimental data at different stages of the simulation, to either guide the search for correct structures or help evaluate the results, in order to combine the reliability of hard data with the convenience of simulations. Herein, the applications of BiGGER are described by illustrative applications divided in three Case Studies: (Case Study A) in which no specific contact data is available; (Case Study B) when different experimental data (e.g., site-directed mutagenesis, properties of the complex, NMR chemical shift perturbation mapping, electron tunneling) on one of the partners is available; and (Case Study C) when experimental data are available for both interacting surfaces, which are used during the search and/or evaluation stage of the docking. This algorithm has been extensively used, evidencing its usefulness in a wide range of different biological research fields.

## 1. Introduction

Protein-protein recognition processes are crucial for a wide range of biological functions, including gene transcription and translation, cell growth, cell differentiation, immune response, and neurotransmission. Recent developments in array technology and high-throughput yeast two-hybrid screens led to an increase in the list of known protein-protein associations [1,2,3,4] and to a growing demand for understanding the structural features of these interfaces to help elucidate kinetic and thermodynamic aspects of complex stabilization and what determines their specificity [5].

Moreover, the obvious importance of protein-protein interactions motivates the design of drugs that can specifically inhibit interactions between relevant proteins [6,7], a task that is considerably harder if the structure of the protein complex is unknown. Even though different strategies can help mitigate this difficulty [8,9], this still leads to a growing demand for docking simulations that can take advantage of all available data.

Protein complexes can be classified depending on their binding affinity into high (K_d_: nM–fM), intermediate (K_d_: μM–nM) or weak (K_d_: mM–μM). Complexes with high and intermediate affinities have long-lifetimes and can be considered static complexes. Examples of these include protease-inhibitor, antigen-antibody, T-cell receptor-peptide-MHC and signal transduction complexes [10,11]. Complexes with a weak affinity (e.g., electron transfer complexes) have short lifetimes on the millisecond time-scale) and are called transient complexes. A short lifetime is important for high turnover rates in redox proteins, often compromising the specificity of the interactions because, though the affinity must be high enough to allow fast electron transfer, it cannot be so high that it prevents the rapid dissociation of the products, which would lower the turnover of the electron transfer chain, for instance [12].

The molecular structure of a protein-protein complex can be difficult to determine by either X-ray crystallography or NMR spectroscopy, especially those with a transient nature. However, molecular docking procedures can be used to obtain a model structure of the complex when the atomic coordinates of the individual proteins are known.

In general, molecular docking involves two steps: global (local) search and scoring [13,14,15,16,17,18,19]. In the first stage, the proteins are usually treated as rigid bodies, and one of them is kept fixed while the other is free to rotate and translate, searching the six-dimensional space for the candidate complexes. These structures are evaluated by a simple scoring function, based mainly on shape complementarity, in order to reduce the number of models to analyze. The second stage scores the candidate models using several parameters, including statistics of residue-residue contacts across interfaces of complexes [20,21], electrostatics, hydrogen bonding, change in accessible solvent area, and lack of buried charges [13]. This scoring function is meant to indicate how well the candidate model corresponds to the real complex.

This approach assumes there are no significant conformational changes upon binding [22]. However, small conformational changes, such as in lysines and arginines that are highly flexible [23], must be accounted for, either during the search stage (“soft” docking) [24] or as an additional refinement using molecular dynamics simulations [25,26,27,28].

Since this approach depends on the possibility of distinguishing correct models from the large number of incorrect models generated, it is less successful with weak interactions. However, experimental data can be used to filter false positives and improve the probability of finding the correct complex, either by constraining the initial search, or as an additional scoring function in the evaluation stage.

This paper will focus on the applications of the soft-docking algorithm BiGGER (Bimolecular complex Generation with Global Evaluation and Ranking) [24], available at [29] with the Chemera modeling application. We intend to illustrate how this program can be used depending on the information that is available on the proteins complexes being modeled. A detailed description of the BiGGER algorithm and possible sources of experimental restraints is provided in the next section, and several case studies are presented grouped in three docking categories: Case A—Docking without any specific experimental data on the interaction surface; Case B—Docking with data on the interaction surface of only one protein or with other experimental data; and Case C—Docking with data on the interaction surface of both proteins. The use of this docking program has been steadily cited since 2000, and has reached in July 2016 a total of about 80 applications, described in 66 publications (Figure S1). A list of most protein complexes predicted by BiGGER is reported in Table S1 with their relative properties.

## 2. Methodology

BiGGER is designed to integrate as much information as available into the docking procedure. Therefore, the search stage is implemented using constraint programming techniques [30] to efficiently search an order of 10^15^ potential configurations resulting from the typical translation and rotation steps of 1 Å and 15°. As is generally the case with protein-protein docking algorithms, the main scoring parameter at this stage is surface contact area. However, BiGGER can additionally constrain the search space in order to model information regarding potential contacts or distances between parts of the proteins. These can come either from experimental data, as some examples in the Case Studies section illustrate, or from other sources, such as assumptions regarding the interaction or even contact predictions [31]. These constraints can range from distance limits between specific atoms to more undefined restrictions such as a minimum number of unspecified contacts between sets of atoms or residues. This versatility enables BiGGER to model a wide range of relevant data, from precise contacts to ambiguous or noisy information, such as that obtained by techniques, like site-directed mutagenesis.

Additional data can also be used to improve the scoring stage, widely recognized as the most difficult step in modeling protein interactions (see, e.g., [14] for a review on scoring methods), especially with transient protein complexes, due to the interplay of many factors involved in protein orientation, recognition and the dynamics of association and dissociation. Although the recent application of machine learning yield promising results in the classification of candidate models [32], transient protein complexes raise the additional problem of being under-represented in the data set of experimentally determined structures, on which scoring functions can be trained and tested. This poses a significant problem because transient complexes are a challenge from a modeling perspective, not only for constituting the majority of the catalytic interactions but also for being the most difficult complexes to determine experimentally and, thus, the category where computer simulations would be most useful. BiGGER makes it easier to use experimental data to score and rank the models, thus improving performance at this stage, whenever such data are available. Furthermore, pruning the search space during the first stage also contributes to improving the results of the scoring stage by improving the ratio of correct models to false positives.

These features stem from a fundamental difference between BiGGER and most protein docking software, in that BiGGER was conceived as a tool to help researchers elucidate protein interactions rather than as an automated predictor. BiGGER participated in round 2 of the Critical Assessment of PRediction of Interactions (CAPRI) program [33,34]. As a blind docking predictor, BiGGER preformed on the same level as most participants. None of 13 participating groups successfully predicted targets 4 and 5, BiGGER and other five predictors, out of 16, proposed good models for target 6 and, for target 7. The best model provided by BiGGER had an interface rmsd of 2.33 Å, placing BiGGER approximately half-way down the ranking of the 15 participants submitting predictions for this target, using this measure. Despite its purpose being the integration of relevant information and helping in the generation of hypotheses [35], blind docking benchmarking has always been an important part of testing BiGGER, from the beginning up to recent innovations in the use of constraints for improving docking results [36,37]. However, blind docking is neither a realistic application nor a very reliable one, regardless of which software one uses. Rather than trusting a black-box predictor, in practice, it is more useful and reliable to allow researchers as much freedom as possible to guide the docking process and use its predictions to help formulate new hypotheses and conceive relevant experiments. This is where BiGGER deviates from the majority of protein docking tools, placing a greater emphasis on flexible integration of information and the analysis of patterns in the results instead of aiming for a blind prediction of a specific model.

### 2.1. Search Stage

**Figure 1:**
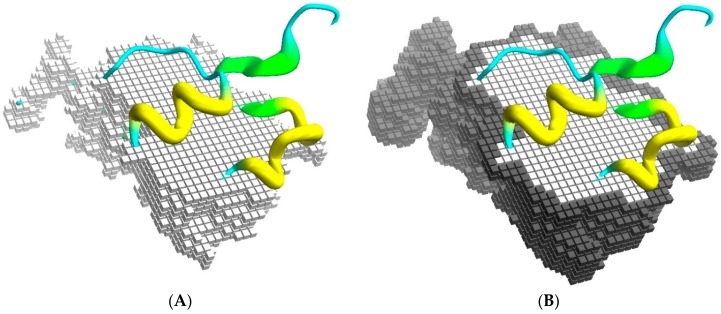
Docking grids. (**A**) Part of the core grid superimposed on the cartoon representation of the Annexin 24 monomer (PDB ID 1dk5) [38]; and in (**B**) is shown the surface cells. The surface contact is scored from the overlap of surface cells between the grid representations of both partners, while the overlap of core regions is not allowed.

BiGGER is designed to take experimental data into account during the search stage by restricting the search space to those configurations that respect some specified constraints. The algorithm is based on a conceptualization of the docking process as a space of potential configurations that is pruned by constraints. The most basic constraints are those that define the scope and goal of the search stage of the docking simulation: prevent excessive overlap between the partners and maximize surface contact, which is achieved by representing the shape of each molecule with two cubic-celled grids, one for the core region of the molecule and one for its surface [24]. Figure 1 illustrates this representation conceptually, although the actual implementation is encoded as sets of linear segments of contiguous grid cells to improve the efficiency of filtering the forbidden collisions of core regions and counting the overlap of surface regions, which determines the surface contact score.

In addition to these fundamental constraints, information about the interaction can be included in the search stage as additional constraints. These can take the form of specified intervals for the placement of one partner relative to the other, distances between atoms, a minimum number of contacts between groups of atoms in both partners or between a group of atoms in one partner and any atom in the other partner [30]. In these latter cases, the contacts themselves do not need to be specified, so the constraints implicitly account for any uncertainty in the data. For instance, if there is evidence from site directed mutagenesis that mutations at ten residues of one partner interfere with the formation of the complex, it is not necessary to assume that all ten residues are at the interface. A reasonable constraint on the docking models could be that at least five of these ten residues be in contact with the other protein, thus accounting for the fact that mutations can interfere with complex formation through means other than by being at the interface. The residues in contact need not be specified, and any five would fulfill the constraint.

In general, the application of BiGGER to real cases has relied on such constraints to restrict or validate the generated models, using data on the reaction centers [39], from NMR spectroscopy [40,41,42] or site-directed mutagenesis [43], as we will show in the case studies.

Taking advantage of this capacity for including generic constraints, our current research includes integrating residue contact predictors based on homologous sequence analysis in BiGGER. Our results show that, with adequate processing, multiple sequence alignments can provide information on potential contacts that can be extracted automatically and used to prune the configuration search space, thus reducing false positives, using the same algorithms BiGGER already uses for integrating experimental data [31].

**Figure 2:**
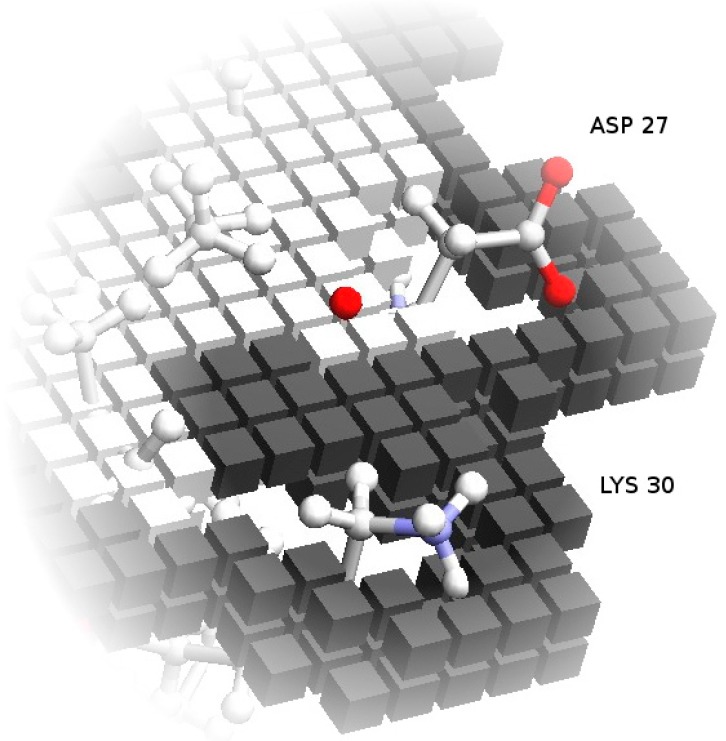
Soft docking grid. Using Annexin 24 monomer (PDB ID 1dk5) [38] as an example, this figure shows the core grid cells removed at the locations corresponding to the exposed side-chains of Asp27 and Lys30. Surface grid cells, represented in a darker color, still conform to the specific configuration of these residues in the PDB file. As an additional detail, note that the surface cells actually define an external shell of the protein shape. This is deliberate, so that the maximum overlap of surface cells from two grids corresponds to a realistic distance between the atoms from the two partners.

One fundamental problem with grid-based docking methods is the assumption that the docking partners are perfectly rigid—with some tolerance for implicit mobility given by the size of the grid, which removes structural details smaller than the grid step. Though this tends to be approximately true for globular proteins, which usually retain the same overall shape when interacting, the exact placement of side chains at the surface can interfere with the formation of the complex. To simulate, implicitly, the mobility of these side-chains, which would change conformation to accommodate the interface contacts, the more mobile exposed side-chains are removed from the core grids. This retains a preference for the native conformation, which determines the surface grids, but prevents these side-chains from excluding correct models due to core-core overlaps (process illustrated in Figure 2).

### 2.2. Scoring Stage

The approach of integrating experimental data in the docking process also applies to the scoring stage. In addition to scoring each model according to surface contact area, side-chains contact likelihoods derived from known structures, solvation estimates and electrostatics (included in the so called Global Score), BiGGER also includes the ability to score models according to parameters, such as number of contacts between specified residues (information derived from NMR chemical shift perturbation mapping, steady-state kinetics or mutagenesis data analysis), minimum distances between specified groups of atoms, symmetry for homodimers, electron transfer estimates (e.g., analysis of the electron tunneling using the software PATHWAYS of the high scored complexes, which is performed *a posteriori* [44,45]), or disordered regions that were identified as potential interacting sites [46,47]. This makes it easy to score and rank the candidate models with relevant empirical data, or to include any additional scoring criteria that may be relevant for the particular complex being modeled. The scoring stage is described in detail in the original reference and in the software documentation.

**Figure 3:**
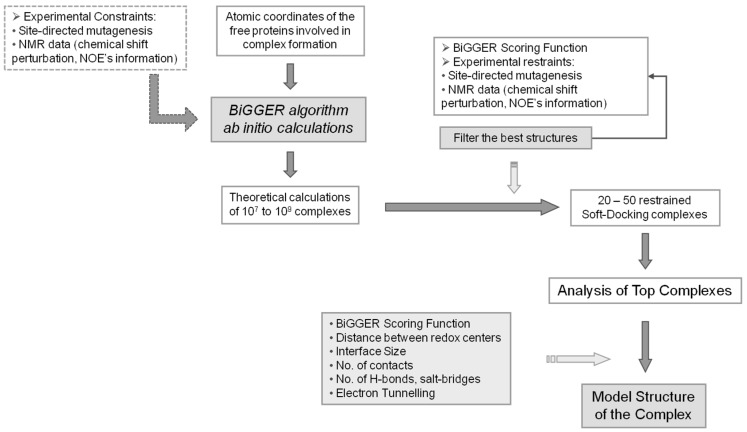
BiGGER Docking Process. Diagram explaining the approach used in molecular docking simulations using BiGGER. The atomic coordinates of the free proteins are entered into the protein docking algorithm [24]. Initial ab initio calculations by BiGGER produce 10^7^ to 10^9^ complexes, which are filtered and scored according to BiGGER Scoring function and 1000 putative docked positions are retained. These complexes are then scored and ranked using experimental restraints (e.g., NMR chemical shift perturbation). The highest ranked complexes are analyzed and a model structure for the complex is obtained. Adapted from Morelli, et al. [48].

A schematic representation of the global procedure that can be followed when using BiGGER is represented in the flow chart of Figure 3.

## 3. Data Analysis

The standard way of evaluating specific aspects of docking algorithms is to use a benchmark of known complexes and assess the success rate of a standardized application of the docking algorithm to the benchmark set. This was also our approach at several stages of the development of BiGGER. For example, for the first evaluation of the algorithm [24]; for performance improvements and assessment [30]; for the application of constraints to restrict the search space [31]. However, the uniform application of a docking algorithm does not accurately capture the way docking is used in practice, especially in the case of BiGGER, whose main goal is to improve the integration of all available data. Since in real applications each complex to model is associated with a unique set of data about the interaction, a more realistic assessment of the usefulness of a docking algorithm is its actual use in real-life applications. As this manuscript focuses on BiGGER as a tool to integrate available data with docking predictions, rather than focusing on any particular aspect of the implementation, we opt to rest the claim to the usefulness of our approach on the set of published papers reporting applications of BiGGER to modeling protein complexes. Although not strictly a docking benchmark, a set of 80 applications to real problems published by several independent groups is a good data set on which to ground claims to the usefulness of a docking algorithm. Given the diversity of data that can be relevant for protein docking, this may even be a better benchmark than a default and uniform application of the algorithm to sets of known complexes.

The next subsections show some examples of filters that BiGGER can use.

### 3.1. Properties of the Complex

The character of the complex can be derived from how the affinity between the two proteins varies with ionic strength using techniques such as isothermal titration microcalorimetry (ITC), Förster resonance energy transfer (FRET), or kinetic assays.

In general, the decrease in the affinity or activity with increasing ionic strength indicates an electrostatic complex [49,50,51,52], while in a hydrophobic complex, the affinity either increases or is not affected at high ionic strength values [53,54]. The knowledge of this property can be used to rank the solutions obtained by BiGGER algorithm by the Hydrophobic or Electrostatic Score, instead of the usual Global Score.

### 3.2. Important Residues in Complex Formation

The residues identified by site-directed mutagenesis or by NMR spectroscopy (2D titrations—chemical shift perturbation mapping) as important for complex formation and/or involved in the interacting surface can be used to either constraint the docking during the search stage or filter the solutions obtained at the evaluation stage of the docking. In the latter case, residues in either the target or the probe that take part in the interface will be considered when ranking the putative solutions, using the shorter distance between those important residues and the partner as a criterion; in the former case, they will be used during the calculation to limit the three-dimensional search, as explained above [40,55,56,57,58] (see Table S1).

In the case of electron transfer complexes, residues that are known to play a crucial role in electron transfer can be used to rank solutions or discard putative complexes, wherein those residues are not placed in the electron transfer pathway (for instance, residues identified using the program PATHWAYS, an analysis that is performed after the docking on the high scored complexes) [44,45].

### 3.3. Distance between Redox Centers

Electron tunneling time scales must be in the millisecond to microsecond range for biological redox machines to function properly. Several theoretical and experimental data show that the maximum center-to-center distance for a fast single-step tunneling through proteins cannot be larger than 20 Å [59,60]. Therefore, in the case of electron transfer complexes, the top solutions can be selected by choosing only the complexes that present appropriate distances between the electron transfer cofactors (distance shorter than 20 Å).

## 4. Case Studies

This section reports several case studies to illustrate how BiGGER has been used to model protein complexes with limited available experimental data on the protein complex (Case Study A); with experimental data on the interacting surface of the probe (Case Study B) or on both the probe and the target (Case Study C).

### 4.1. Case A—Docking without or Little Filtering

The Electron Transfer Complex between Aldehyde Oxido-Reductase (AOR) and flavodoxin, a transient complex, was modeled using BiGGER to test the hypothesis that this complex mimics the domains/subunits arrangement of other similar proteins [49]. This case study can be regarded as a limit situation to the application of this docking algorithm. The 3D structures of the proteins that belong to the Xanthine Oxidase family show different solutions for the problem of transferring electrons between the flavin adenine nucleotide (FAD) group and the molybdenum site, where the substrate is handled. In Xanthine Oxidase, four redox centers are present in a single polypeptide chain. The intra-molecular electron transfer is promoted from the Mo site to the two [Fe-S] centers and on to FAD, where it is transferred to an electron acceptor/donor component.

**Figure 4:**
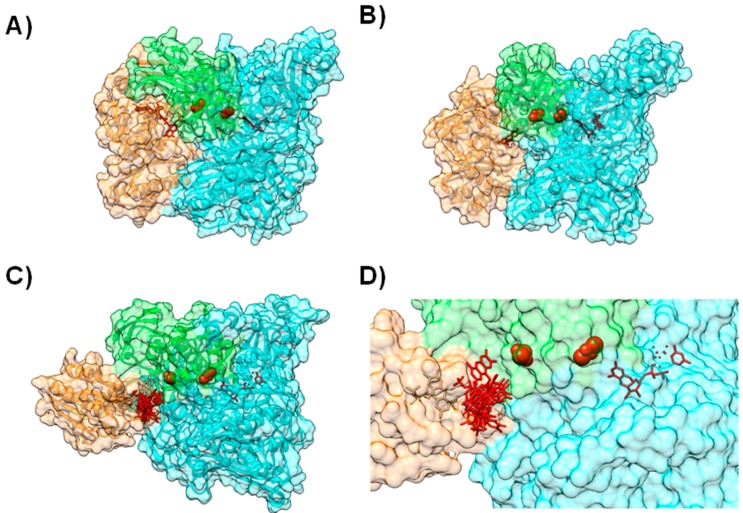
Modeling AoR-Flavodoxin complex. Cofactor arrangement on Xanthine Oxidase (**A**); CO dehydrogenase (**B**) and Aldehyde Oxido-Reductase (**C**,**D**), with each domain or subunit colored accordingly. Panels (**C**) and (**D**) depict the result for the docking of *D. gigas* Flavodoxin to Aldehyde Oxido-Reductase; (**C**) Structure of the highest ranking model and the placement of the FMN group in the 10 top ranking models; (**D**) A close up on the redox centers. In all the panels the [Fe-S] binding domain is represented in green, the Mo-binding domain in cyan and the FMN or FAD binding domains in orange. Figure prepared using UCSF Chimera [69].

In other proteins from this molybdenum family, the cofactors have a similar organization but are distributed within different subunits (Figure 4), as can be observed by comparing the structures of Xanthine Oxidase (XO) [61] (Figure 4A), CO-Dehydrogenase (COD) [62] (Figure 4B), Xanthine Dehydrogenase (XDH) [63], AOR [64,65,66] and 4-Hydroxylbenzoyl-CoA Reductase (4-HBCR) [67,68]. For instance, the [Fe-S] centers, FAD and the Mo-pterin cofactors of COD are bound to three different polypeptide chains and the enzyme exists as a stable complex of three different subunits (α, β, and γ) (Figure 4B).

In the case of AOR, the Mo-pterin cofactor and the two [Fe-S] centers are present within a single polypeptide chain and no flavin component has been detected [70]. However, an electron flow deriving from aldehyde oxidation was demonstrated in vivo, involving the Mo active center, the [Fe-S] centers, and a flavin component (an FMN group of flavodoxin). This process was shown to be essential for the continuity of the intermolecular electron transfer to cytochrome *c*_3_ and onwards to Hydrogenase, yielding a final net production of hydrogen [70] (an example with hydrogenase will be presented in Case B, Example 2). Since, this electron transfer pathway involves 11 discrete redox centers, the complex between AOR and flavodoxin must not only be essential, but also resemble the “entire” redox assembly depicted by Xanthine Oxidase and its family members [49].

In order to model this protein complex, BiGGER was used without any additional altering or constraints. The authors used the available X-ray structure of *Desulfovibrio* (*D.*) *gigas* AOR (PDB ID 1HLR) [65,66] and the homology models for both *D. gigas* and *D. salexigens* flavodoxins, obtained using the 3D structure of *D. vulgaris* flavodoxin (PDB ID 1FX1) [71] as template, in two separate docking studies (at the time its tridimensional structure was not known, PDB ID 4HEQ [72]). Solutions were ranked according to the electrostatic energy minimization score, and model structures were identified and validated by comparison with other known structures of the enzymes from the XO family. These different dockings helped validate the docking of *D. gigas* AOR with flavodoxin (Figure 4C,D).

The final outcome predicted that both flavodoxins interact with AOR near the more exposed [Fe-S] center in a configuration that resembles the structural arrangement of XO and the homologous [Fe-S] in the single polypeptide chain (Figure 4C,D). Moreover, the modeled complexes replicate the structural arrangements and architecture of redox sites of not only XO, but also of COD and 4-HBCR [49] (Figure 4).

We believe this is a good example for the versatile solutions found by Nature in the construction of complex electron transfer chains and another exploratory method for the use of the BiGGER algorithm in situations where no constraints can be imposed a priori.

### 4.2. Case B—Solutions Filtered by Experimental Data

**Figure 5:**
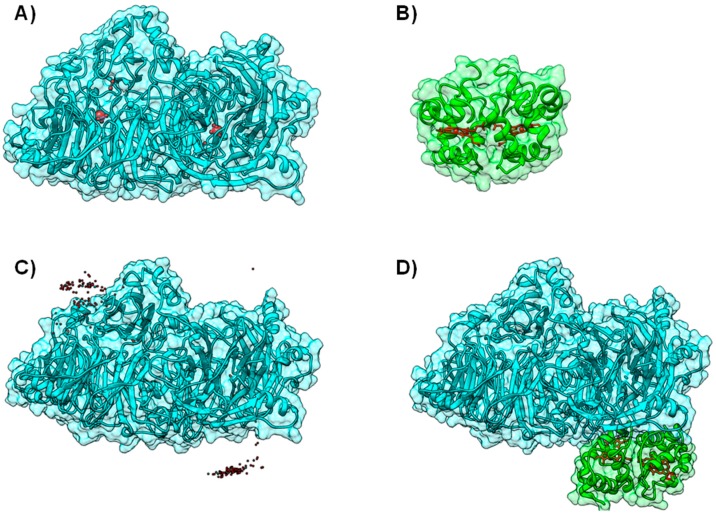
Electron transfer complex between Nitrous Oxide Reductase and cytochrome *c*_552_ from *Marinobacter hydrocarbonoclasticus*. Panels (**A**) and (**B**) depict the backbone structure of *Marinobacter hydrocarbonoclasticus* N_2_OR and cytochrome *c*_552_, respectively. Metal centers (CuA and CuZ center for N_2_OR and heme for cytochrome *c*_552_) are represented in red; Panel (**C**) 200 top docking solutions ranked by hydrophobic score of N_2_OR—cytochrome *c*_552_ electron transfer complex. The iron atom in the heme of each cytochrome *c*_552_ putative docking position is represented as a red sphere; Panel (**D**) A top model structure for the electron transfer complex obtained by BiGGER is represented (probe 2, Table S1 [54]). N_2_OR is colored cyan and cytochrome *c*_552_ is colored green. Figure prepared using UCSF Chimera [69].

A summary of cases in which BiGGER was used in conjunction with experimental data can be found in Table S1 of the Supplementary Materials. One example was the modeling of Nitrous Oxide Reductase (N_2_OR). This enzyme catalyzes the last step of the denitrification pathway, the reduction of nitrous oxide to molecular dinitrogen, which provides an electron sink for some bacterial species to grow under anaerobic conditions [73,74]. N_2_OR has two multi-copper centers located in two different structural domains: a binuclear electron transfer center, CuA center, and a tetranuclear copper center, CuZ center, the catalytic site. The large distance between CuA and CuZ centers within the same monomer imposes a dimeric conformation on the enzyme, which is thus a functional homodimer, in which the two subunits are oriented “head to tail”, bringing CuA and CuZ centers to approximately 10 Å, an appropriate distance for an efficient electron transfer (Figure 5A).

The study presented in [75] used BiGGER to obtain model structures of the electron transfer complexes between nitrous oxide reductase from *Marinobacter hydrocarbonoclasticus* (PDB ID 1QNI) [76], *Paracoccus denitrificans* (PDB ID 1FWX) [77], *Achromobacter cycloclastes* (PDB ID 2IWF) [78], and their respective physiological electron donor(s) (PDB IDs 1CNO, 1COT and 3ERX, and 1BQR, respectively). Ionic strength dependence of the mediated nitrous oxide reduction was the experimental data source used at the scoring stage. In the case of *Marinobacter hydrocarbonoclasticus*, cytochrome *c*_552_ [79] (Figure 5B) is the physiological electron donor and the interaction between the two proteins is mainly hydrophobic [54], while electron transfer complexes involving N_2_OR from other bacterial sources have an electrostatic character.

In the former particular case, the docking results agreed well with the experimental data, as for *Marinobacter hydrocarbonoclasticus* N_2_OR, a high number of solutions with appropriate orientation and distance between cofactors for electron transfer (below 20 Å) was found just by ranking the docking solution using the Hydrophobic Score [54,75] (Figure 5C,D). On the other hand, *Paracoccus denitrificans* cytochrome *c*_550_ shows strong “affinity” towards the CuA surface area of N_2_OR in both hydrophobic and electrostatic rankings. A similar result was obtained with *Paracoccus pantotrophus* and *Achromobacter cycloclastes* pseudoazurins and the enzyme of the same organism, even if this small electron carrier shows a lower number of putative complexes due to the smaller surface interacting with N_2_OR, when compared to that of the *c*-type cytochromes studied [75].

In fact, it has been proposed that both electrostatic and hydrophobic interactions play an important role in the formation of electron transfer complexes [80,81,82], as long-range recognition is mainly driven by electrostatic forces to form the encounter complexes, and hydrophobic interactions fine-tune the formation of the electron transfer complex.

**Figure 6:**
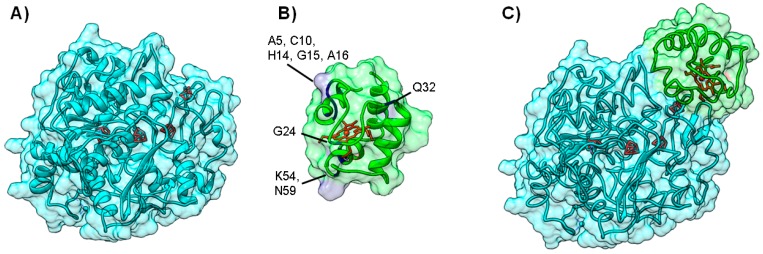
Electron transfer complex between Hydrogenase and cytochrome *c*_553_ from *D. desulfuricans*. Panels (**A**) and (**B**) depict the backbone structure of Hydrogenase and cytochrome *c*_553_, respectively. Metal cofactors ([4Fe-4S] clusters and binuclear iron H cluster for Hydrogenase and *c*-type heme for cytochrome *c*_553_) are represented in red. Grey colored residues in cytochrome *c*_553_ are the most affected in a ^1^H-^15^N HSQC NMR titration, and are clearly marked on the picture Panel (**C**) Top model structure for the electron transfer complex obtained combining NMR and soft-docking (PDB ID 1E08) [40]. Hydrogenase surface is colored cyan, while cytochrome *c*_553_ surface is colored green. Figure prepared using UCSF Chimera [69].

The second example is the electron transfer complex between cytochrome *c*_553_ and Fe-Hydrogenase, in which models were filtered using NMR Data. Molecular hydrogen plays a central role in the metabolic activity of several sulfate-reducing bacteria, in particular in *Desulfovibrio* sp., where it can be used as a source of electrons and energy [83,84]. Hydrogenase catalyzes both the splitting and the formation of the hydrogen molecule [85,86]. The structure of *D. desulfuricans* Hydrogenase, an iron-sulfur enzyme (PDB ID 1HFE) [87], shows the presence of two subunits of 42 kDa and 10 kDa, respectively. The large subunit has a ferredoxin-like domain with two [4Fe-4S] clusters (one of which located close to the negatively-charged protein surface) and a second domain with a single [4Fe-4S] cluster bridged to the active site, which is constituted by a binuclear iron center, named H cluster, buried within the protein core (Figure 6A) [87].

Based on a kinetic study [88], the electron donor of Fe-Hydrogenase was proposed to be the monohemic cytochrome *c*_553_ (Figure 6B), a periplasmic, low potential cytochrome *c* analogue to the mitochondrial cytochrome *c* [89]. In this case, the NMR restrained docking approach was useful to filter docking solutions [40]. In a typical procedure, the small electron carrier protein, cytochrome *c*_553_ (PDB ID 1DVH) [90], was isotopically labeled with ^15^N and changes in the ^1^H-^15^N TROSY HSQC spectrum [91] of cytochrome *c*_553_ were followed during the course of a titration. Upon complex formation, it was possible to identify residues that are involved in the complex interface (Chemical Shift Perturbation Mapping) [92]. In particular, eight H^N^ groups of the residues affected by complex formation have been used to filter the docking solutions. The remaining solutions were ranked according to the level of agreement with the NMR constraints. Of the top 50 solutions, 10 models exhibit a heme/H cluster distance smaller than 20 Å.

The energy-minimized calculation of the 10 resulting solutions allowed the authors to choose one of the model complexes as the best representative structure of the complex (Figure 6C). This solution (Fam1 solution 3) [40], which has a short heme/H cluster distance (12.3 Å), obeys 5 of the NMR constraints, and residues K63 and Y64 are present at the interface. These residues are essential for electron transfer, as indicated by site-directed mutagenesis [93].

### 4.3. Case C—Information on the Interacting Surface from Both Partners

**Figure 7:**
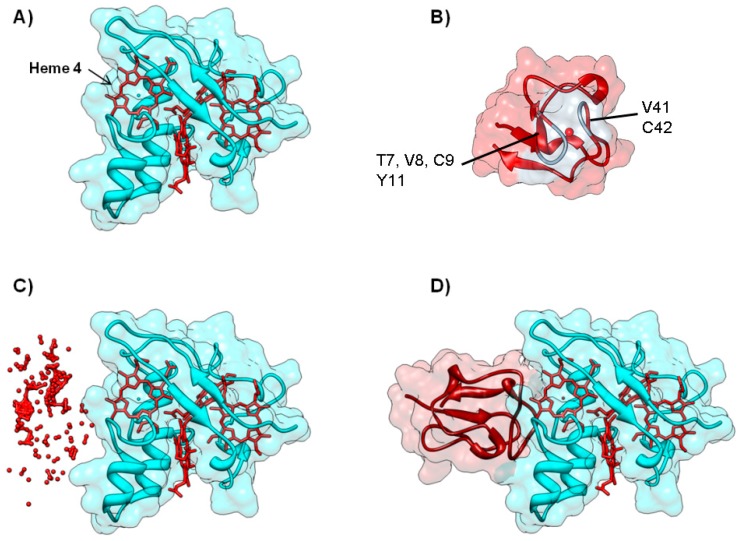
Electron transfer complex between cytochrome *c*_3_ and rubredoxin from *D. gigas*. Panels (**A**) and (**B**) depict the backbone structure of the cytochrome *c*_3_ and rubredoxin, respectively. Metal centers (*c*-type heme for cytochrome *c*_3_ and Fe for rubredoxin) are represented in red. Rubredoxin residues whose ^N^H resonances are most affected in a ^1^H-^15^N HSQC NMR titration are shown in light grey; Panel (**C**) The top 200 docking solutions ranked by electrostatic energy minimization score of the cytochrome *c*_3_—rubredoxin electron transfer complex. The iron atom of each rubredoxin putative docking position is represented as a red sphere; Panel (**D**) Top model structure for the electron transfer complex obtained by BiGGER is represented. Cytochrome *c*_3_ is colored cyan and rubredoxin is colored dark red. Figures prepared using UCSF Chimera [69].

There are several reports in the literature in which the docking solutions are filtered using experimental information on both interacting surfaces. One such case is the interaction between cytochrome *c*_3_ and rubredoxin from *D. gigas* [94]. The former is a conserved tetrahemic *c*-type cytochrome present in the periplasm of sulfate reducing bacteria (Figure 7A), involved in the hydrogen metabolism and electron transfer processes to periplasmic and membrane-bound multi-heme cytochromes in metabolic pathways that ultimately generate ATP [95]. Rubredoxin is a small (5.7 kDa) metalloprotein containing a FeS_4_ center, present in the cytoplasm of different types of anaerobic and aerobic bacteria (Figure 7B); among several proposed interactions, *D. gigas* rubredoxin is thought to transfer electrons to enzymes involved in oxygen detoxification pathways: superoxide reductase and rubredoxin:oxygen oxidoreductase, in order to regenerate their catalytic metal centers after superoxide or oxygen reduction, respectively [96].

Both proteins present characteristic NMR paramagnetic effects: the presence of four low-spin heme groups in cytochrome *c*_3_ induces contact and pseudo-contact shifts on heme methyls that generate chemical shifts in ^1^H NMR spectra that lie on the so-called “paramagnetic region” (10–35 ppm). These well-resolved resonances can serve as region-specific probes that help identify interaction surfaces.

Likewise, the presence of a high-spin Fe^3+^ in rubredoxin’s metal center induces a series of paramagnetic effects in NMR spectra, the most significant of which consisting of Paramagnetic Relaxation Enhancements (PREs) brought upon by its five unpaired electrons creating a “blind zone” around the Fe ion with a well-known distance dependence (see ref. [97] for more information). However, rubredoxin presents another significant advantage: its metal center can easily be replaced with Zn^2+^ (among other metals) which is of special importance for NMR, as Zn^2+^ is diamagnetic, which in turn allows the observation of resonances close to the metal center.

Although cytochrome *c*_3_ and rubredoxin do not interact in vivo due to their localization in different cellular compartments, they can still serve as probes for the interaction between heme proteins and [Fe-S] cluster-containing proteins. BiGGER was used in this case as a validation and prediction tool in order to characterize the most likely complex geometry according to NMR-derived experimental data. Thus, upon acquisition and analysis of 2D NMR [^1^H-^15^N HSQC] titration spectra, it was possible to identify the regions involved in the interaction by chemical shift perturbation mapping. Residues near the metal center of Zn-substituted rubredoxin (T7, V8, C9, Y11, V41 and C42) were found to suffer the largest perturbations [94]. Concomitantly, the most exposed methyl resonances belonging to heme IV suffered specific and distinct line width broadenings ascribed to the proximity of rubredoxin’s high-spin Fe^3+^ iron-sulfur cluster (not observed in a titration using Zn-rubredoxin).

These sets of data were then used as ab initio constraints in the docking calculations using BiGGER. The Fe ion of rubredoxin was placed at a distance shorter than 4 Å from heme IV’s methyls M2 and M18 of cytochrome *c*_3_ (Figure 7A,D). In addition, by calculating the line width broadening ratio and using the relevant Solomon-Bloembergen-Morgan equations, the authors established that the rubredoxin Fe-cytochrome *c*_3_ M18 distance should be 1.3 times larger than the one between rubredoxin Fe and cytochrome *c*_3_ M2 (Figure 7D). BiGGER was successful in generating models for the electron transfer complexes that could verify all the constraints imposed a priori. For the cytochrome *c*_3_:rubredoxin complex, the model that was obtained places the Fe atom of rubredoxin within 5 Å of the exposed heme IV methyls, consistent with the observed PRE effects (Figure 7D). The residues predicted to be at the complex interface are also consistent with those whose chemical shift suffered the largest perturbations in the NMR titration experiments.

The same approach was used to characterize the complex between pseudoazurin (PAz) and copper-containing Nitrite Reductase (NiR) from *Alcaligenes faecalis* S-6 [41]. PAz is 14 kDa protein containing a type 1 copper center, and is responsible for shuttling electrons to NiR’s active site. The latter protein is a 110 kDa homotrimer, containing 6 copper atoms, and is the enzyme responsible for an important step in the denitrification pathway, the reduction of nitrite to nitric oxide [74]. The active sites (three per protein) comprise one copper atom in a type 2 Cu center, while the remaining copper atoms are buried within the protein’s core in a type 1 geometry. Enzymatic assays of several site-directed mutants have established that exchanging four of the thirteen lysine residues of pseudoazurin (K10, K38, K57 and K77) into either alanine or aspartate led to a decrease in binding affinity, but without concomitant decrease in the reaction rate [98,99].

In the NiR-pseudoazurin interaction, Paz residues K109, H81, H40, A15, M84, I110, M16, Y82, A83, K10, K107, R114 and V17 were described as suffering the largest chemical shift perturbations in a NMR titration [41]. In this case, during the docking calculation, the constraint was set so that 8 of the 13 most-perturbed pseudoazurin residues would be in contact with any surface residue of NiR. In a similar way to Example 1, the distance between the copper atoms of pseudoazurin and the NiR type 1 copper atom was determined to be 14 Å, which is within the limit for direct electron transfer between the metal centers. Furthermore, residues (both charged and uncharged) having the greatest chemical shifts variations in the NMR titration were placed at the complex interface in the top docking models.

## 5. Discussion and Final Remarks

Protein-protein complexes have an essential role in many metabolic pathways and discovering their structure is an important step in the development of strategies to interfere with protein interactions. However, determining the structure of protein-protein complexes remains a difficult task, especially if the complex or the individual proteins are not very stable and the short life-time of the complex does not allow co-crystallization. This is most often the case for complexes associated with high turn-over processes, such as electron transfer. While molecular docking is generally a useful and inexpensive complement to studying protein interactions, in these cases it becomes a crucial part in inferring plausible structures from limited data.

In general, protein docking algorithms aim to predict the structure of a complex from the structure of the partners with the assumption that there is one correct structure for the complex, as seems to be the case for proteins that co-crystallize in stable configurations. BiGGER differs from most docking software in both its goal and some fundamental assumptions. BiGGER does not assume that the interaction results in a single, stable complex, and thus includes visualization and computation tools, such as clustering, to help analyze families of models that may represent more dynamic and labile complexes. Furthermore, the main goal of BiGGER is to help integrate relevant data, by either restricting the search space or screening candidate structures, in order to test more general hypotheses regarding the interaction other than one presumed unique structure, such as preferred binding sites, active prosthetic groups, stoichiometry and so forth. Because of this approach, BiGGER is not fine-tuned towards stable complexes, such as those that co-crystallize and for which it is possible to compare the predicted complex with a unique structure. As a result, BiGGER trades some accuracy, relative to other popular docking algorithms and automated servers, when it is used to predict stable complexes using only the structures of the partners. In return, BiGGER provides a more flexible framework for dealing with transient complexes for which no single structure may be fully representative of the interaction or when additional information is available, such as site-directed mutagenesis or spectroscopic data.

Of the popular docking algorithms, HADDOCK (envisaged by Alexandre Bonvin and co-workers) [27,100,101] is perhaps the most similar to BiGGER in that it is also designed to use experimental data to guide predictions and help evaluate families of potential candidates. However, HADDOCK is mostly used with NMR data, which may be either an advantage or disadvantage depending on the available data. Furthermore, HADDOCK requires these data for the definition of ambiguous interaction restraints (AIRs) that can drive the prediction, whereas in BiGGER additional data is optional.

Finally, BiGGER is meant to be easily available, with a low computation cost, and thus does not include model refinement stages for more precise predictions, nor does it automate the search through different conformations of the interacting partners. This is generally not a problem when modeling weak interactions between globular proteins, where the atomic resolution of the models may not be meaningful and conformational change is negligible, but may be a drawback when modeling stronger complexes between flexible partners.

Some authors have used in their work both BiGGER and HADDOCK. Although their mode of operation is slightly different, they have found that the algorithms calculated very similar results from the same initial data [102,103]. In both cases, complex orientation was refined with NMR chemical shift perturbation mapping data [*ab initio* in HADDOCK, and as a model filtered in BiGGER]. One other interesting case where both algorithms were used can be found in [104], wherein the authors used BiGGER (along with ClusPro) on an initial stage in order to create interaction restraints to be used in HADDOCK.

The results available, especially from test cases in real applications, show that BiGGER can provide useful interaction information by integrating a variety of experimental data from different sources, either during the search or the evaluation stages after running the algorithm. The evaluation and validation of the structural model of the protein complex is the most important stage, and it should be performed with care, using all the experimental data available. To this end, BiGGER allows the use of different types of filters to restrain the solutions, such as the properties of the encounter complex (i.e., hydrophobic or electrostatic character), identification of important residues in complex formation and the distance between redox centers (if present).

The case studies presented included examples with limited experimental data or with experimental data on one or both interacting surfaces to show how BIGGER can be used to integrate any available information at different stages of the docking simulations, either as constraints that can guide the predictions or as scoring functions to validate the candidate models. Even when no experimental information is available (Case study A), the docking program can help design mutants or develop other strategies to better understand the protein complex by providing well defined hypotheses regarding the domains involved at the interface.

BiGGER has also an important role in the validation and corroboration of experimental evidence regarding a protein-protein interaction (Case studies B and C). In this case, the algorithm prediction helps make use of the experimental data by finding the structures that are consistent with the information available. In addition, we would like to point out that the final model complex(es) can be energy minimized, which can be performed using software such as UCSF Chimera [69], in order to remove side-chain clashes created by the docking procedure.

These results give an overview of the protein-protein algorithm BIGGER, which has been extensively used by biochemical and biological community since the year 2000, as shown by the different case studies that illustrate the potentiality of the algorithm in both prediction and validation of a protein-protein complex. Moreover, the case studies give detailed examples of the procedure for protein docking and the analysis of the results, providing a helpful guide for non-specialist users.

## Supplementary Materials

Supplementary materials can be accessed at: http://www.mdpi.com/1420-3049/21/8/1037/s1.
