# Supplementary material for: Predicting Protein-Protein Interactions Using BiGGER: Case Studies
Source: Molecules. 2016 Aug 9;21(8):1037. doi: 10.3390/molecules21081037 (PMC6274584; doi:10.3390/molecules21081037)
Supplement: Supplementary file 1 [file molecules-21-01037-s001.pdf]

Supplementary Materials: Predicting Protein-Protein Interactions Using BiGGER. Case Studies

Rui M. Almeida, Simone Dell'Acqua, Ludwig Krippahl, José J. G. Moura and Sofia R. Pauleta

Table S1. List and relative properties of protein-protein complexes predicted by BiGGER algorithm since year 2000.

Paper Ref	PDB ID	Protein Name	Method	Year	Restraints?	Filtering?	Nature of Complex	Other Exp. Methods	Kd (Order of Magnitude)?	Any Homology Modelling?
Ali et al., (2014) [1]	Scorpium neurotoxins Agiotoxin 2 (1AGT), kaliotoxin (2KTX), kaliotoxin 2 (1KTX), OsK1 (1SCO), BmKTx1 (1BKT), and K+-channel KcsA (1BL8). Homology models of Bs-KTx6 and mKv1.3	Agiotoxin 2, kaliotoxin, kaliotoxin 2, OsK1, BmKTx1, KcsA	BiGGER, Electrophysiological studies	2014	No	No	N/A	Electrophysiological studies	N/A	Yes, with MODELLER
Almeida et al., (2009) [2]	1WAD for cytochrome c_3, 1RDG for rubredoxin, both from *Desulfovibrio gigas*	Rubredoxin, cytochrome c_3 from *Desulfovibrio gigas*	BiGGER, NMR	2009	No	Yes, NMR	Electrostatic	NMR	25 micromolar	No
Amela et al., (2013)	2GA5 for Yfh1, homology models for Isu and Nfs1, *ab initio* model calculated for Isd11	Yeast Frataxin Homolog 1, Isu, Nfs1 and Isd11 from yeast	BiGGER, EscherNG, Hex, HADDOCK	2013	No	No	Undisclosed	EscherNG, Hex, HADDOCK	No	ESyPred3D, 3D-PSSM, Phyre, Rosetta
Andreotti et al., (2005) [3]	1BL8 (KScA channel), for modeling; 1PNH, 1SCY and 1TXM	sKCa2, sKCa3 and Scorpion Toxins P05, Lei and MTX	BiGGER	2005	No	Yes—comparison of Kd values with docking energies	Electrostatic	Kd values	Yes—nM range	INISIGHTII Discover 3 module, MODELLER, NMRCLUST
Banci et al., (2003) [4]	N/A	Cytochrome b_5 from rabbit, iso-1-cytochrome *c* from yeast	BiGGER, NMR	2003	Yes - NMR	NMR, atom contacts	Electrostatic	NMR, Energy minimization of best structures	N/A	Sander module on AMBER 6.0 for energy minimization
Bauer et al., (2013) [5]	4HPK and 3JQW for *holo* s3b (ColG) and *holo* s3 (ColH) from *Clostridium histolyticum*, 1K6F for collagenous peptide	*Clostridium histolyticum* ColH and ColG collagen binding domains, Collagen.	BiGGER, X-Ray Crystallography, SAXS, Fluorescence Spectroscopy	2013	No	No	N/A	X-Ray Crystallography, SAXS, Fluorescence Spectroscopy	No	MIFit
Bonding et al., (2008) [6]	Horse heart cytochrome *c* (1HRC), 1OJ6 for neuroglobin	Horse heart cytochrome *c*, neuroglobin	BiGGER, NMR, Surface Plasmon Resonance (SPR)	2008	Not used in docking	Heme Fe-Fe distance no more than 15 Angstrom	Electrostatic	NMR, SPR	200 µM, by NMR, 20 to 125 µM by SPR	YASARA back-modelling of neuroglobin

Bustos and Iglesias (2005) [7]	1EUH, for modeling of five plant GAPN proteins; 1A4O	Phosphorylated non-phosphorylating glyceraldehyde-3-phosphate dehydrogenase (GAPN), 5 plant species; 14-3-3	SS Kinetics, BiGGER	2005	Yes - selected by user, based on previous results with model peptide	AA Distance filtering (S404 from GAPN, K39 + R56 + R127 from 14-3-3	Electrostatic	SS Kinetics of NADPH formation (reaction is NADP + dependente)	Kinetic rates only	Yes, for GAPN using Modeller v6.0; Energy minimization with AMBER force field in HyperChem 5.0
Cai et al., (2003) [8]	*Ab initio* structure calculations	Caveolin-1, Several SARS-CoV proteins with caveolin binding motifs	BiGGER	2003	No. Selected model with lowest energy	Lowest energy		No	No	All caveolin binding proteins were modeled with SWISSPROT
Carpentier et al., (2014) [9]	1HPN, 1FNH	the standard dodecasaccharide ([IdUA2S-GlcNS6S]6) of heparin; Hep-II domain of fibronectin (1FNH)	Bioinformatics (potential energies of interaction)	2014	No	Yes	Variable	Docking software: GRAMM, ESCHER, HEX (rigid docking). GOLD (flexible)	N/A	No
Carrega et al., (2005) [10]	1QUZ (HsTx1), 1Y2P ([Abu^{19},Abu^{34}]-HsTx1], 1WZ5 ([Abu^{20},Abu^{35}]-Pi1), 1ORQ to generate models of human calcium channels	S5-H5-S6 pore regions of mKv1.1, rKv1.2, and mKv1.3 (human calcium channels); Scorpion toxins HsTx1 and Pi1	BiGGER, NMR, Electrophisiology	2005	Best complexes were energy minimized using the GROMOS96 force field on Deepview 3.7	No	Not clear, but strong	NMR, Electrophisiology, Energy minimizaation	N/A	Human calcium channels modeled with CNS
Chapon et al., (2000) [11]	N/A	*Erwinia chrysanthemi* CD_{Cel5} and CBD_{Cel5} (3 domains)	BiGGER, Mutagenesis studies, Secretion studies (Western Blot)	2000	Post-docking selection of Trp43 distance to catalytic domain	Amino Acid Distance filtering	Not clear	Mutagenesis, Secretion Studies	N/A	N/A
Cozza et al., (2007) [12]	Bovine RNase A (1F0V, 1A2W, 1JS0, and homology models)	Oligomers of Bovine RNase A, both native and domain-swapped;	BiGGER; Zdock, Gramm, Escher	2007	None	RMSD comparison with published structures	Not clear	Other docking studies	N/A	Yes
Crowley et al., (2002)a [13]	*Phormidium luminosum* cytochromes *c* and *f* (1CI3, 1YCC); spinach Rieske protein (1RFS)	*Phormidium luminosum* cytochrome *f* and cytochrome *c*; Rieske protein from spinach	BiGGER, NMR	2002	Yes, Chemical shift perturbation mapping	NMR restraints	Electrostatic	NMR	50–250 μM	No
Crowley et al., (2002)b [14]	N/A	*Phormidium laminosum* cytochrome f; cytochrome c_6 from *Anabaena* sp. PCC 7119 and *Synechococcus elongatus*	BiGGER, NMR	2002	No	Yes, NMR	Electrostatic	NMR chemical shift perturbation mapping	100 μM and higher	No

Cruz-Gallardo et al., (2013) [15]	1JZG for azurin, 1NIN for plastocyanin, 1A3Z for rusticyanin	Merozoite Surface Protein 1, 19 kDa C-terminal fragment, and *Acidithiobacillus ferrooxidans* Rusticyanin, *Nostoc* sp. PCC 7119, *Phormidium laminosum* and poplar plastocyanins, and *Pseudomonas aeruginosa* azurin (Az)	BiGGER, NMR, ITC, Culture Growth Inhibition	2013	Yes, for MSP-1:Rc complex	Yes, NMR	Mainly hydrophobic	NMR, ITC, Culture Growth Inhibition	2 micromolar	No
Czjzek et al., (2002) [16]	*Desulfovibrio vulgaris* cytochrome c_3 (2CTH); *ab initio* structural determination of Hmc (1GWS)	Hexadeca-heme cytochrome Hmc, cytochrome c_3 from *D. vulgaris*	Bigger, Crystallography, NMR	2002	Not at first	Yes, NMR	Electrostatic	NMR CSPM, Xtal	No	No
Dell'Acqua et al., (2008) [17]	*Marinobacter hydrocarbonoclasticus* N_2OR (1QNI) and cytochrome c_{552} (1CNO), horse heart cytochrome *c* (1HRC)	*Marinobacter hydrocarbonoclasticus* N_2OR and cytochrome c_{552}, horse heart cytochrome *c*	BiGGER, Enzymatic Activity assays, NMR	2008	No	Cluster to cluster distance	Electrostatic	NMR, Activity Assays	5 µM	No
Dell'Acqua et al., (2011) [18]	*Marinobacter hydrocarbonoclasticus* N_2OR (1QNI), *Paracoccus denitrificans* N_2OR (1FWX), *A. cycloclastes* N_2OR (2IWF), *Marinobacter hydrocarbonoclasticus cytochrome* c_{552} (1CNO), horse heart cytochrome *c* (1HRC), *Paracoccus denitrificans cytochrome* c_{550} (1COT), *Paracoccus pantotrophus* pseudoazurin (3ERX), *A. cycloclastes* pseudoazurin (1BQR), bovine heart cytochrome *c* (2B4Z)	Several related to N_2OR and its electron donors from multiple organisms	BiGGER	2011	No	No	Electrostatic	Several Bioinformatics tools	N/A	Yes, using PHYRE and SWISSMODEL
deMorree et al., (2011) [19]	CAPN3 modelled from 3DF0, 2T7P (Filamin C)	CAPN3 (Calpain 3), FLNC (Filamin C)	BiGGER, Bioinformatics	2011	No	No	N/A	*Ab initio* bioinformatics	No	SWSSMODEL for CAPN3
El Antak et al., (2003) [20]	*Desulfovibrio vulgaris* hydrogenas (2HFE), cytochrome c_3 (2CTH)	cytochrome c3, Fe-hydrogenase from *Desulfovibrio vulgaris*	BiGGER, NMR, MD simulations	2003	No	Yes, NMR and cluster to cluster distance	Electrostatic	NMR, Molecular Dynamic Simulations to minimize energy with X-PLOR	N/A	No
Fantuzzi et al., (2009) [21]	*Desulfovibrio vulgaris* Flavodoxin (1J8Q); *Bacillus megaterium* P450 BMP (1BU7)	*Desulfovibrio vulgaris* Flavodoxin and *Bacillus megaterium* P450 BMP	BiGGER, NMR, Electron Transfer studies	2011	No	Yes, NMR	Electrostatic	NMR, Electron Transfer	tens to hundreds micromolar	Yes

Fujita et al., (2012) [22]	*Achromobacter cycloclastes* pseudoazurin (1BQR, 1BQK) and Nitrous Oxide Reductase (2IWF); *Marinobacter hydrocarbonoclasticus* cytochrome c_{552} (1CNO) and N_2OR (1QNI). Bovine heart cytochrome *c* (2B4Z)	*Achromobacter cycloclastes* pseudoazurin and Nitrous Oxide Reductase; *Marinobacter hydrocarbonoclasticus* cytochrome c_{552} and N_2OR. Bovine heart cytochrome *c*.	BiGGER, Kinetic assays, Electrochemistry, Western Blot	2012	Energy minimization using Tinker 4.2	Distance between clusters	N/A	Kinetic assays, Electrochemistry, Western Blot	N/A	Yes, energy minimizations using Tinker 4.2. Best complexes were analyzed for ET using PATHWAYS
Giron-Monzon et al., (2004) [23]	*E. coli* MutL (1B63), and MutH (2AZO)	*E.coli* ATPase MutL and Endonuclease MutH	BiGGER, Cross-Linking, site-Directed Mutagenesis	2004	No	4 angstrom cutoff, comparison with cross linked models	Dependent on DNA presence	Mutagenesis, Cross-linking	N/A	Comparison with models derived from cross-linking
Impagliazzo et al., (2005) [24]	*Alcaligenes faecalis* Pseudoazurin (8PAZ) and Nitrate reductase (1AQ8)	Pseudoazurin and Nitrate Reductase from *Alcaligenes faecalis*	BiGGER, NMR	2005	NMR-derived	No	Electrostatic	NMR Chemical shift perturbation mapping	N/A	No
Impagliazzo et al., (2010) [25]	Peptide derived from epitope of polyadenylate binding protein nuclear 1 (PABPN1), modelled by PHYRE, and llama antibody 3F5 (homology model based on 1G9E)	Polyadenylate binding protein nuclear 1 (PABPN1) and llama antibody 3F5	BiGGER, NMR, Fluorescence, Stopped-Flow Kinetics	2010	No	No	Mostly hydrophobic	NMR, Fluorescence, Stopped-Flow Kinetics	0.8 uM	Yes, with PHYRE and SWISS-MODEL
Jokiranta et al., (2006) [26]	Human C3d (1C3D), FH19-20 (2G7I), homology modelling, 1RID (heparin)	Human FH19–20 with C3d and heparin	BiGGER, Crystallography, Affinity Column, Radioligand assay	2006	No	Exclusion of solutions binding to other known binding site	N/A	Crystallography, Affinity, Radioligand	N/A	Yes
Jonker et al., (2005) [27]	*Herpes simplex* PC4ctd dimer (2PHE), homology models of VP16ad α-helices, and TFIIBc	*Herpes simplex* PC4ctd dimer, VP16ad R-helices, and TFIIBc	BiGGER, HADDOCK, NMR, Site-directed mutagenesis	2005	No	Energy minimization of best complexes with GROMOS96 force field	Electrostatic	NMR, SDM	N/A	Yes, SWISSMODEL for TFIIBc and VP16ad α-helices
Jouirou et al., (2004) [28]	*Centruroides noxius* NMR-generated structure of cobatoxin 1, *in silico*-generated peptides	*Centruroides noxius* CoTX1 or ACoTX1 with human Kv1.2 Ca^{2+} channel	BiGGER, NMR, Molecular Dynamics simulations	2004	No	Geometric Filtering of Top 5 solutions	Geometric	NMR, MD	Yes—nM	Yes—peptides
Kovaleski et al., (2007) [29]	Capsid CA chain P (1E6J); homology model of human LysRS using 1E1O	HIV CA Capsid Protein; Human Lysil-tRNA synthase	BiGGER, Site-directed mutagenesis	2007	4Å cut-off	Comparison with deletion mutants	Strong (770 nM)	Spectrofluorimetry	Yes. By spectrofluorimetry	Yes, using MODELLER 4

Krippahl et al., (2006) [30]	*Desulfovibrio. Vulgaris*, *D. gigas* and *D. salexigens* aldehyde oxidoreductase (1HLR and homology models), and Flaxodoxin (1FX1 and homology models)	Aldehyde Oxidoreductase and Flavodoxin from *D. vulgaris*, *D. gigas* and *D. salexigens*	BiGGER	2006	No	Metal cluster to metal cluster distance	Transient	No	N/A	Yes
M'Barek et al., (2003)a [31]	Scorpion Maurotoxin (1TXM), Homology models of rat potassium channels (from 1BL8)	Scorpion Mautoxin MTXPi1 and the various voltage-gated K channel subtypes (Drosophila Shaker B, rat Kv1.1 Kv1.2, and Kv1.3 S5-H5-S6 domains)	BiGGER, Ecotoxicity	2003	No	15 best from global score, Toxin Lys and beta-sheet orientation, electrostatics	Strong	Ecotoxicity	N/A	Yes, using INSIGHTII, PROCHECK, SWISSMODEL
M'Barek et al., (2003)b [32]	Homology model of rat calcium channels (from 1BL8), and scorpio toxin sPi4 (1N8M)	Scorpio toxin sPi4, rat Kv1.2 calcium channel s5-H5-S6 fragment	BiGGER, NMR, Electrophisiology, EC_{50} studies	2003	No	Top five scorers	Strong	Electrophisiology, NMR, EC_{50}	N/A	Yes, using INSIGHTII, PROCHECK, SWISSMODEL
M´Barek et al., (2005) [33]	Scorpion butantoxin-Maurotoxin chimera (1WT7); homology model of S5-H5-S6 pore region of Kv1.2 channel (1ORQ)	BuTX-MTXPi1 (together as a chimera, as well as individually; Kv1.2 Channel from rat	BiGGER, Circular Dichroism, NMR, Electrophisiology	2005	No	Yes: global score, relative orientation of probe to target, and likeliness of electrostatic interactions	N/A	NMR, Electrophisiology, Modeling, Energy minimization	N/A	Yes, SWISSPROT and INSIGHTII
Martinez-Fabregas, et al.(2014) [34]	1J3S, 4ALD, 2RR6, 1Q8K, 2E50, 2BR9, and homology models	Human cytochrome *c*, ALDOA, ANP32B, eIF2α , hnRNP C1/C2, HSPA5, SET, STRAP, and YWHAE	BiGGER, Affinity Chromatography, MALDI-TOF/TOF, Bimolecular fluorescence complementation, Surface Plasmon Resonance, NMR, ITC	2014	No	No	N/A	Affinity Chromatography, MALDI-TOF/TOF, Bimolecular fluorescence complementation, Surface Plasmon Resonance, NMR, ITC	N/A	Yes, with MODELLER
Matamala et al., (2007) [35]	*Fremyella diplosiphon* phycocyanin hexamer (1CPC)	Phycocyanin hexameric form from *Fremyella diplosiphon*	BiGGER, Crystallography, FRET	2007	No	20 best of each parameter.	Geometric	FRET, Crystallography	N/A	No
McKenna et al., (2003) [36]	Human hUbc13hMms2 heterodimer (1J74); Human Ubiquitin (1UBQ)	Human Ubiquitin; and hUbc13 hMms2 heterodimer.	BiGGER, NMR Chemical Shift Perturbation	2003	No	Filtered by Chemical shift perturbation mapping, imposing upper distance limit of 5Å	Electrostatic	NMR	100 micromolar, determined earlier	Yes, best complexes were energy-minimized using INSIGHTII

Monaco et al., (2007) [37]	Human Matrix Metalloproteinase 2 (1CK7); for Bovine fibrinogen (1M1J).	Truncated forms of human MMP2, Bovine Fibrinogen	BiGGER, Activity assays, zymography	2007	N-terminal to C-terminal of probe no longer than 20 angstrom	No	Electrostatic	Zymography, activity assays	N/A	Models of fibrinogen with amino acid deletions were also modelled
Monini et al., (2012) [38]	Homology models of TAT BH10 and gp120 domain ΔV1-2 sf162	TAT BH10 and gp120 domain ΔV1-2 sf162	BiGGER, ClusPro, HADDOCK, isothermal Calorimetry Titration, Site-directed Mutagenesis, Infection Experiments, Flow Cytometry, Confocal Microscopy, Electron Microscopy, Surface Plasmon Resonance, ELISA	2012	No	*A posteriori* refinement of initial results using other docking algorithms	Strong	ClusPro, HADDOCK, isothermal Calorimetry Titration, Site-directed Mutagenesis, Infection Experiments, Flow Cytometry, Confocal Microscopy, Electron Microscopy, Surface Plasmon Resonance, ELISA	111 nM	Yes, Modeller 8
Morelli 2000 JBC [39]	1DVH for *Desulfovibrio vulgaris* cytochrome c_{553}, 1HFE for Fe-Hydrogenase from *Desulfovibrio desulfuricans*	*Desulfovibrio vulgaris* cytochrome c_{553}, Fe-Hydrogenase from *Desulfovibrio desulfuricans*	BiGGER, NMR, MD Energy Minimization using X-PLOR and SHAKE	2000	No	Yes, NMR	Electrostatic	NMR, Molecular Dynamics Energy Minimization	N/A	Yes, Greenpath to predict tunneling pathway
Morelli 2000 Biochem [39]	1DVH for *Desulfovibrio vulgaris* cytochrome c_{553}, homology model of Ferredoxin I from *Desulfomicrobium norvegicum*	*Desulfovibrio vulgaris* cytochrome c_{553}, Ferredoxin I from *Desulfomicrobium norvegicum*	BiGGER, NMR, MD Energy Minimization using X-PLOR	2000	No	Yes, NMR	Electrostatic	NMR, Molecular Dynamics Energy Minimization	N/A	Yes, with TURBO-FRODO and SHAKE
Morelli et al., (2001) [40]	*Escherichia coli* Enzyme I (1ZYM) and HPR (1POH), *Bacillus amyloliquefaciens* Barnase (1A2P) and barstar (1A19), Rat Tom20 (1OM2), Yeast cytochrome *c* (1CCP) and cytochrome *c* peroxidase (1YCC)	Several test complexes (EIN + HPr; Barnase + Barstar; Tom20 + Presequence; cytochrome *c* + cytochrome *c* peroxidase)	BiGGER, NMR	2001	NMR restraints (Chemical Shift Perturbation, Amide Proton-Deuterium Exchange rates)	No	Transient to Strong	NMR	Variable, from μM to pM	Yes
Mouhat et al., (2004) [41]	Rat Kv channels models (from 1KVC); peptides	Pi1, [A24,A33]-Pi1 or P-Pi1 on to rat Kv 1.1, Kv1.2 and Kv1.3 Ca^{2+} channels	BiGGER, Conformational Analysis, electrophysiology	2004	No	Take five best scorers, take to Turbo-Frodo	Strong (low values of IC50)	NMR, Electrophisiology	Strong (nM range)	Yes, all Kv channels, using Turbo-Frodo, Procheck, Whatif and CNS

Nishida et al., (2003) [42]	Human von Willebrand factor A3 domain (1AO3); shortened model peptide of collagen (2CLG); microfibril shortened model (4CLG)	Human A3 domain, collagen peptide, microfibril	BiGGER; NMR, Surface Plasmon Resonance, Site-Directed Mutagenesis	2003	No	NMR filtering	Transient	NMR, Surface Plasmon Resonance, Site-Directed Mutagenesis	Micromolar range	Yes
Nummelin et al., (2004) [43]	Modified collagen-like peptide (1CAG); Model of *Yersenia enterocolitica* YadA (1P9H)	*Yersenia enterocolitica* YadA, other Peptides (including collagen)	BiGGER, Crystallography, Site-directed mutagenesis	2004	No	NMR filtering; max. distance 4 or 5 angstrom	Transient to strong	Crystallography, Site-directed mutagenesis	Micromolar range	Yes, using SOLVE and ArpWarp
Palma et al., (2005) [44]	*Synechocystis* sp. PCC 6803 Ferredoxin-NADP$^+$ Reductase modeled from 1QUE; Ferredoxin modeled from 1QT9	*Synechocystis* sp. PCC 6803 Ferredoxin-NADP$^+$ Reductase and Ferredoxin	BiGGER, NMR	2005	No	NMR and Electron Tunneling pathway filtering	Transient	NMR	N/A	Yes, with SWISSMODEL
Pauleta et al., (2004) [45]	*Paracoccus pantotrophus* pseudoazurin (1ADW)	*Paracoccus pantotrophus* cytochrome *c* Peroxidase (monomer) and pseudoazurin	BiGGER, NMR, Isothermal Calorimetry Titration, Analytical Ultracentrifugation	2004	No	NMR filtering	Transient	NMR, Isothermal Calorimetry Titration, Analytical Ultracentrifugation	low micromolar	No
Pedroso et al., (2016) [46]	Homology model based on 1HZV, 1CNO, 1HRC	Cytochrome cd1NiR, cytochrome c552, horse heart cytochrome c	BiGGER, Cyclic voltammetry	2016	No	Yes	Hydrophobic	Cyclic voltammetry	N/A	Yes, with I-TASSER
Pettigrew et al., (2003)a [47]	N/A	*Paracoccus denitrificans* cytochrome *c* peroxidase monomer with two horse heart cytochrome *c* molecules	BiGGER, Analytical Ultracentrifugation, Isothermal Calorimetry	2003	No	No	Transient	Analytical Ultracentrifugation, Isothermal Calorimetry	very low micromolar	No
Pettigrew et al., (2003)b [48]	N/A	*Paracoccus denitrificans* cytochrome *c* peroxidase and cytochrome c_{550}, horse heart cytochrome *c*	BiGGER, Analytical Ultracentrifugation, Isothermal Calorimetry, NMR	2003	No	Yes, NMR	Transient	Analytical Ultracentrifugation, Isothermal Calorimetry, NMR	Low micromolar	No
Philominathan et al., 2009 [49]	1NQD and 1K6F for *Clostridium histolyticum* Collagen Binding Domain and collagenous peptide, respectively	CBD and collagenous peptide from *Clostridium histolyticum*	BiGGER, NMR Chemical Shift Perturbation, Steady-state fluorescence, Circular Dichroism, SAXS	2009	No	From NMR results	N/A	NMR Chemical shift perturbation mapping, Steady-state fluorescence, Circular Dichroism, SAXS	57 μM	Yes
Philominathan et al., (2012) [50]	*Clostridium histolyticum* collagenase (1NQD) and minicollagen (1CAG)	Collagenase and Minicollagen from *Clostridium histolyticum*	BiGGER, NMR, Small Angle X-ray Scattering, Circular Dichroism	2012	No	From NMR results	N/A	NMR, Small Angle X-ray Scattering, Circular Dichroism	N/A	No
Pieulle et al., (2004) [51]	*Desulfovibrio africanus* Pyruvate-ferredoxin oxidoreductase (1B0P) and ferredoxin I (1FXR)	Pyruvate-ferredoxin oxidoreductase; ferredoxin I from *D. africanus*	BiGGER, Isothermal Titration Calorimetry, NMR, Electrochemistry, Activity assays	2004	No	Yes, NMR	Transient	Isothermal Titration Calorimetry, NMR,electrochemistry, Activity assays	tens of micromolar	No

Pieulle et al., (2005) [52]	*Desulfovibrio alaskensis* Type I and II cytochrome *c_3* (2BQ4 and 3CAO); *Desulfovibrio vulgaris* Type I and II cytochrome *c_3* (2CTH and a homology model)	Type I and II cytochrome *c_3* from *Desulfovibrio alaskensis* and *D. vulgaris*	BiGGER, Analytical Ultracentrifugation, Isothermal Calorimetry, Cross-linking	2005	No	Yes	Transient	Analytical Ultracentrifugation, Isothermal Calorimetry, cross-linking	very low micromolar	Yes, MODELLER 6.0 for type II cytochrome *c_3* from *D. vulgaris*
Poletto et al., (2008) [53]	Human CK2 (1JWH)	Human CK2, one peptide	BiGGER, Phosphorilation assays, ZDOCK, GRAMM, Escher	2008	No	Comparison between Docking algorithms	N/A	Phosphorylation, other docking algorithms	Km values only	Yes, energy of best complex minimized using AMBER99
Rajalingam 2005 [54]	Human fibroblast growth factor and amlexanox	Human FGF-1and antibiotic amlexanox	BiGGER, ITC, NMR, Turbidometry	2005	No	Yes, NMR	Transient	Turbidometry, NMR, ITC	80 μM	Yes, energy of best complex minimized using AMBER99
Raychaudhuri et al., (2010) [55]	N/A	cytochrome c, neuroglobin	BiGGER, PCR, Western, Flow cytometry	2010	No	No	Transient	Flow cytometry, Western, PCR	tens of micromolar	N/A
Ribeiro Jr. et al., (2009) [56]	Phosphoprotein P (1VYI) and N_{11}-RNA complex (2GTT)	Phosphoprotein P and Nucleoprotein N from rabies virus	BiGGER, SPR, SAXS, SANS	2009	No	No	Strong	SPR, SAXS, SANS	160 nM	Yes, with LOBO, SYMMDOCK, EMPIRE
Rudolph et al., (2007) [57]	Human Yjef_N model (from 1JZT), human apolipoprotein A1 (1AV1)	Human AI-BP and its homologues hYjeF_N2-15q23 and hYjeF_N3-19p13.11; Apolipoprotein A1	BiGGER, PCR, Northern Blotting, Western Blotting, Immunocytochemistry	2007	No	No	N/A	PCR, Northern Blotting, Western Blotting, Immunocytochemistry	N/A	Yes, using Permol v. 1.01 and Modeller v 6.2
Saglietti et al., (2007) [58]	Human Glutamate Receptor Glu R2 (modeled from 1EWK, 1JDP, and 1DP4)	Human Glutamate Receptor GluR2-N Terminal Domain and N-Cadherin-Ecto	BiGGER, Immunocytochemical assays (varied)	2007	Constraint to AA 14-92	Yes	N/A	Immunocytochemistry	N/A	Yes, with mGenthreader Modeller, Verify3D, SWISSMODEL
Skommer and Brittain (2012) [59]	Human cytochrome *c* (1HRC) and neuroglobin (1OJ6)	Human cytochrome *c* and neuroglobin	BiGGER, *in silico* and site-directed mutagenesis, RT-PCR, Flow Cytometry	2012	No	Yes, Fe-Fe distance	N/A	*in silico* and site-directed mutagenesis, RT-PCR, Flow Cytometry	N/A	Yes, *in silico* mutations on neuroglobin using YASARA and energy minimization using the AMBER99 force field
Spies and Kowalczykowski (2006) [60]	*Escherichia coli* RecA (2REB) and RecB (1W36)	*E.coli* RecA and $RecB^{nuc}$	BiGGER, Ni-NTA Magnetic beads	2007	No	Comparison with crystal of larger hetero-mer	Transient to Moderate	Ni-NTA Magnetic Beads	1 micromolar	Yes

Srinivasan et al., (2004) [61]	Mouse CD80 model (Templates: 1DR9, 1NCN, 118L and 1I85); Mouse CD152 model (from 1DQT, 1AH1)	Mouse CD80 + CD80-CAP	BiGGER, Proliferation assays, Circular Dichroism, ELISA	2004	No	TOP 100 superimposed on known structure	Strong	Circular Dichroism, ELISA, Proliferation Assays	N/A	Yes, using Geno3D. Energy minimization using GRAMMOS
Winkler et al., (2009) [62]	N/A	*Chlamydomonas reinhardtii* FeFe hydrogenase HydA1 and Photosynthetic ferredoxin PetF	BiGGER, Steady State Kinetics, Site-directed mutagenesis	2009	No	Cluster to cluster distance, electrostatics, mutagenesis studies	Electrostatic	Steady State Kinetics, Site-directed mutagenesis	N/A	Yes, using SWISSMODEL
Worrall et al., (2002) [63]	Horse myoglobin (1HMB), Bovine cytochrome *b*$_5$ (1EHB)	Horse heart Myoglobin and bovine cytochrome *b*$_5$	BiGGER, NMR (Titration and Relaxation)	2002	Residues with highest chemical shift perturbations within 5 Å of target	No	Transient	NMR	Micromolar range	No
Xu et al., (2009) [64]	*Synechocystis* sp. PCC 6803 Ferredoxin:Thioredoxin Reductase (1DJ7) and Thioredoxin (1FB6)	*Synechocystis* sp. PCC 6803 Ferredoxin:Thioredoxin Reductase, Thioredoxin	BiGGER, NMR, HADDOCK	2009	Paramagnetic restraints divide residues into three classes of interaction	No	Transient	NMR, HADDOCK	N/A	No
Yabukarski et al., (2016) [65]	Vesicular Stomatitis Virus Nucleocapsid protein N and viral phosphoprotein P and several deletion mutants of each (taken from 3PMK, 3HHZ)	Vesicular Stomatitis Virus Nucleocapsid protein N and viral phosphoprotein P and several deletion mutants of each	BiGGER, Size-exclusion chromatography coupled with MALLS and Refractometry (RI), NMR, SAXS, SANS, Molecular Dynamics Simulations	2016	No	Yes, amino acid residue distances	Strong	Size-exclusion chromatography coupled with MALLS and Refractometry (RI), NMR, SAXS, SANS, Molecular Dynamics Simulations	60 nM (upper limit)	Yes, with Coot
Zhu et al., (2004) [66]	Human p23 (1EJF) and ATP- or ADP-bound Hsp90 (1AM1 and 1BYQ, respectively)	Human Hsp90 and p23	BiGGER, Genetic Analysis (ET)	2004	No	No, but compared to known structures	N/A	Evolutionary genetics	N/A	No

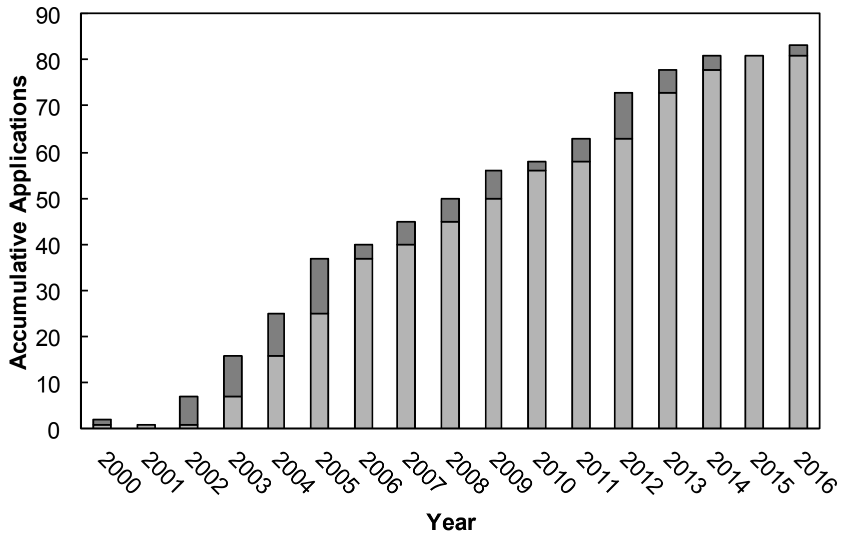

Figure S1. Number of accumulative applications of BiGGER algorithm to predict protein complexes since the year 2000. The light grey segment indicates the number of new applications that have used BiGGER on that year.

References

1. Ali, S.; Alam, M.; Abbasi, A.; Kalbacher, H.; Schaechinger, T.; Hu, Y.; Zhijian, C.; Li, W.; Voelter, W. Structure–activity relationship of a highly selective peptidyl inhibitor of kv1.3 voltage-gated k+-channel from scorpion (b. Sindicus) venom. *Int. J. Pept. Res. Ther.* **2014**, *20*, 19–32.
2. Almeida, R.M.; Pauleta, S.R.; Moura, I.; Moura, J.J.G. Rubredoxin as a paramagnetic relaxation-inducing probe. *J. Inorg. Biochem.* **2009**, *103*, 1245–1253.
3. Andreotti, N.; di Luccio, E.; Sampieri, F.; De Waard, M.; Sabatier, J.M. Molecular modeling and docking simulations of scorpion toxins and related analogs on human skca2 and skca3 channels. *Peptides* **2005**, *26*, 1095–1108.
4. Banci, L.; Bertini, I.; Felli, I.C.; Krippahl, L.; Kubicek, K.; Moura, J.J.G.; Rosato, A. A further investigation of the cytochrome b(5)-cytochrome c complex. *J. Biol. Inorg. Chem.* **2003**, *8*, 777–786.
5. Bauer, R.; Wilson, J.J.; Philominathan, S.T.; Davis, D.; Matsushita, O.; Sakon, J. Structural comparison of colh and colg collagen-binding domains from clostridium histolyticum. *J. Bacteriol.* **2013**, *195*, 318–327.
6. Bonding, S.H.; Henty, K.; Dingley, A.J.; Brittain, T. The binding of cytochrome c to neuroglobin: A docking and surface plasmon resonance study. *Int. J. Biol. Macromol.* **2008**, *43*, 295–299.
7. Bustos, D.M.; Iglesias, A.A. A model for the interaction between plant gapn and 14–3-3 zeta using protein-protein docking calculations, electrostatic potentials and kinetics. *J. Mol. Graph. Model.* **2005**, *23*, 490–502.
8. Cai, Q.C.; Jaing, Q.W.; Zhao, G.M.; Guo, Q.; Cao, G.W.; Chen, T. Putative caveolin-binding sites in sars-cov proteins. *Acta Pharmacol. Sin.* **2003**, *24*, 1051–1059.
9. Carpentier, M.; Denys, A.; Allain, F.; Vergoten, G. Molecular docking of heparin oligosaccharides with hep-ii heparin-binding domain of fibronectin reveals an interplay between the different positions of sulfate groups. *Glycoconj. J.* **2014**, *31*, 161–169.
10. Carrega, L.; Mosbah, A.; Ferrat, G.; Beeton, C.; Andreotti, N.; Mansuelle, P.; Darbon, H.; De Waard, M.; Sabatier, J.M. The impact of the fourth disulfide bridge in scorpion toxins of the alpha-ktx6 subfamily. *Proteins Struct. Funct. Bioinform.* **2005**, *61*, 1010–1023.
11. Chapon, V.; Simpson, H.D.; Morelli, X.; Brun, E.; Barras, F. Alteration of a single tryptophan residue of the cellulose-binding domain blocks secretion of the erwinia chrysanthemi cel5 cellulase (ex-egz) via the type ii system. *J. Mol. Biol.* **2000**, *303*, 117–123.
12. Cozza, G.; Moro, S.; Gotte, G. Elucidation of the ribonuclease a aggregation process mediated by 3d domain swapping: A computational approach reveals possible new multimeric structures. *Biopolymers* **2008**, *89*, 26–39.

13. Crowley, P.B.; Rabe, K.S.; Worrall, J.A.R.; Canters, G.W.; Ubbink, M. The ternary complex of cytochrome f and cytochrome c: Identification of a second binding site and competition for plastocyanin binding. *Chembiochem* **2002**, *3*, 526–533.
14. Crowley, P.B.; Diaz-Quintana, A.; Molina-Heredia, F.P.; Nieto, P.; Sutter, M.; Haehnel, W.; de la Rosa, M.A.; Ubbink, M. The interactions of cyanobacterial cytochrome c(6) and cytochrome f, characterized by nmr. *J. Biol. Chem.* **2002**, *277*, 48685–48689.
15. Cruz-Gallardo, I.; Diaz-Moreno, I.; Diaz-Quintana, A.; Donaire, A.; Velazquez-Campoy, A.; Curd, R.D.; Rangachari, K.; Birdsall, B.; Ramos, A.; Holder, A.A.; et al. Antimalarial activity of cupredoxins: The interaction of plasmodium merozoite surface protein 119 (msp119) and rusticyanin. *J. Biol. Chem.* **2013**, *288*, 20896–20907.
16. Czjzek, M.; ElAntak, L.; Zamboni, V.; Morelli, X.; Dolla, A.; Guerlesquin, F.; Bruschi, M. The crystal structure of the hexadeca-heme cytochrome hmc and a structural model of its complex with cytochrome c(3). *Structure* **2002**, *10*, 1677–1686.
17. Dell'Acqua, S.; Pauleta, S.R.; Monzani, E.; Pereira, A.S.; Casella, L.; Moura, J.J.G.; Moura, I. Electron transfer complex between nitrous oxide reductase and cytochrome c(552) from pseudomonas nautica: Kinetic, nuclear magnetic resonance, and docking studies. *Biochemistry* **2008**, *47*, 10852–10862.
18. Dell'Acqua, S.; Moura, I.; Moura, J.J.G.; Pauleta, S.R. The electron transfer complex between nitrous oxide reductase and its electron donors. *J. Biol. Inorg. Chem.* **2011**, *16*, 1241–1254.
19. De Morree, A.; Hulsik, D.L.; Impagliazzo, A.; van Haagen, H.H.H.B.M.; de Galan, P.; van Remoortere, A.; t Hoen, P.A.C.; van Ommen, G.B.; Frants, R.R.; van der Maarel, S.M. Calpain 3 is a rapid-action, unidirectional proteolytic switch central to muscle remodeling. *PLoS ONE* **2010**, *5*, doi:10.1371/journal.pone.0011940.
20. ElAntak, L.; Morelli, X.; Bornet, O.; Hatchikian, C.; Czjzek, M.; Alain, D.A.; Guerlesquin, F. The cytochrome c(3)-[fe]-hydrogenase electron-transfer complex: Structural model by nmr restrained docking. *FEBS Lett.* **2003**, *548*, 1–4.
21. Fantuzzi, A.; Meharenna, Y.T.; Briscoe, P.B.; Guerlesquin, F.; Sadeghi, S.J.; Gilardi, G. Characterisation of the electron transfer and complex formation between flavodoxin from d-vulgaris and the haem domain of cytochrome p450 bm3 from b-megaterium. *Biochim. Biophys. Acta Bioenerg.* **2009**, *1787*, 234–241.
22. Fujita, K.; Hirasawa-Fujita, M.; Brown, D.E.; Obara, Y.; Ijima, F.; Kohzuma, T.; Dooley, D.M. Direct electron transfer from pseudoazurin to nitrous oxide reductase in catalytic n2o reduction. *J. Inorg. Biochem.* **2012**, *115*, 163–173.
23. Giron-Monzon, L.; Manelyte, L.; Ahrends, R.; Kirsch, D.; Spengler, B.; Friedhoff, P. Mapping protein-protein interactions between mutl and muth by cross-linking. *J. Biol. Chem.* **2004**, *279*, 49338–49345.
24. Impagliazzo, A.; Krippahl, L.; Ubbink, M. Pseudoazurin-nitrite reductase interactions. *Chembiochem* **2005**, *6*, 1648–1653.
25. Impagliazzo, A.; Tepper, A.W.; Verrips, T.C.; Ubbink, M.; van der Maarel, S.M. Structural basis for a pabpn1 aggregation-preventing antibody fragment in opmd. *FEBS Lett.* **2010**, *584*, 1558–1564.
26. Jokiranta, T.S.; Jaakola, V.-P.; Lehtinen, M.J.; Parepalo, M.; Meri, S.; Goldman, A. Structure of complement factor h carboxyl-terminus reveals molecular basis of atypical haemolytic uremic syndrome. *EMBO J.* **2006**, *25*, 1784–1794.
27. Jonker, H.R.A.; Wechselberger, R.W.; Boelens, R.; Folkers, G.E.; Kaptein, R. Structural properties of the promiscuous vp16 activation domain. *Biochemistry* **2005**, *44*, 827–839.
28. Jouirou, B.; Mosbah, A.; Visan, V.; Grissmer, S.; M'Barek, S.; Fajloun, Z.; Van Rietschoten, J.; Devaux, C.; Rochat, H.; Lippens, G.; et al. Cobatoxin 1 from centruroides noxius scorpion venom: Chemical synthesis, three-dimensional structure in solution, pharmacology and docking on k+ channels. *Biochem. J.* **2004**, *377*, 37–49.
29. Kovaleski, B.J.; Kennedy, R.; Khorchid, A.; Kleiman, L.; Matsuo, H.; Musier-Forsyth, K. Critical role of helix 4 of hiv-1 capsid c-terminal domain in interactions with human lysyl-trna synthetase. *J. Biol. Chem.* **2007**, *282*, 32274–32279.
30. Krippahl, L.; Palma, P.N.; Moura, I.; Moura, J.J.G. Modelling the electron-transfer complex between aldehyde oxidoreductase and flavodoxin. *Eur. J. Inorg. Chem.* **2006**, 3835–3840, doi:10.1002/ejic.200600418.
31. M'Barek, S.; Lopez-Gonzalez, I.; Andreotti, N.; di Luccio, E.; Visan, V.; Grissmer, S.; Judge, S.; El Ayeb, M.; Darbon, H.; Rochat, H.; et al. A maurotoxin with constrained standard disulfide bridging—Innovative strategy of chemical synthesis, pharmacology, and docking on k+ channels. *J. Biol. Chem.* **2003**, *278*, 31095–31104.

32. M'Barek, S.; Mosbah, A.; Sandoz, G.; Fajloun, Z.; Olamendi-Portugal, T.; Rochat, H.; Sampieri, F.; Guijarro, J.I.; Mansuelle, P.; Delepierre, M.; et al. Synthesis and characterization of pi4, a scorpion toxin from pandinus imperator that acts on k+ channels. *Eur. J. Biochem.* **2003**, *270*, 3583–3592.
33. M'Barek, S.; Chagot, B.; Andreotti, N.; Visan, V.; Mansuelle, P.; Grissmer, S.; Marrakchi, M.; El Ayeb, M.; Sampieri, F.; Darbon, H.; et al. Increasing the molecular contacts between maurotoxin and kv1.2 channel augments ligand affinity. *Proteins Struct. Funct. Bioinform.* **2005**, *60*, 401–411.
34. Martinez-Fabregas, J.; Diaz-Moreno, I.; Gonzalez-Arzola, K.; Janocha, S.; Navarro, J.A.; Hervas, M.; Bernhardt, R.; Velazquez-Campoy, A.; Diaz-Quintana, A.; de la Rosa, M.A. Structural and functional analysis of novel human cytochrome c targets in apoptosis. *Mol. Cell. Proteom.* **2014**, *13*, 1439–1456.
35. Matamala, A.R.; Almonacid, D.E.; Figueroa, M.F.; Martinez-Oyanedel, J.; Bunster, M.C. A semiempirical approach to the intra-phycocyanin and inter-phycocyanin fluorescence resonance energy-transfer pathways in phycobilisomes. *J. Comput. Chem.* **2007**, *28*, 1200–1207.
36. McKenna, S.; Moraes, T.; Pastushok, L.; Ptak, C.; Xiao, W.; Spyracopoulos, L.; Ellison, M.J. An nmr-based model of the ubiquitin-bound human ubiquitin conjugation complex mms2 center dot ubc13—The structural basis for lysine 63 chain catalysis. *J. Biol. Chem.* **2003**, *278*, 13151–13158.
37. Monaco, S.; Gioia, M.; Rodriguez, J.; Fasciglione, G.F.; Di Pierro, D.; Lupidi, G.; Krippahl, L.; Marini, S.; Coletta, M. Modulation of the proteolytic activity of matrix metalloproteinase-2 (gelatinase a) on fibrinogen. *Biochem. J.* **2007**, *402*, 503–513.
38. Monini, P.; Cafaro, A.; Srivastava, I.K.; Moretti, S.; Sharma, V.A.; Andreini, C.; Chiozzini, C.; Ferrantelli, F.; Cossut, M.R.; Tripiciano, A.; et al. Hiv-1 tat promotes integrin-mediated hiv transmission to dendritic cells by binding env spikes and competes neutralization by anti-hiv antibodies. *PLoS ONE* **2012**, *7*, e48781.
39. Morelli, X.; Czjzek, M.; Hatchikian, C.E.; Bornet, O.; Fontecilla-Camps, J.C.; Palma, N.P.; Moura, J.J.; Guerlesquin, F. Structural model of the fe-hydrogenase/cytochrome c553 complex combining transverse relaxation-optimized spectroscopy experiments and soft docking calculations. *J. Biol. Chem.* **2000**, *275*, 23204–23210.
40. Morelli, X.J.; Palma, P.N.; Guerlesquin, F.; Rigby, A.C. A novel approach for assessing macromolecular complexes combining soft-docking calculations with nmr data. *Protein Sci.* **2001**, *10*, 2131–2137.
41. Mouhat, S.; Mosbah, A.; Visan, V.; Wulff, H.; Delepierre, M.; Darbon, H.; Grissmer, S.; De Waard, M.; Sabatier, J.M. The 'functional' dyad of scorpion toxin pi1 is not itself a prerequisite for toxin binding to the voltage-gated kv1.2 potassium channels. *Biochem. J.* **2004**, *377*, 25–36.
42. Nishida, N.; Sumikawa, H.; Sakakura, M.; Shimba, N.; Takahashi, H.; Terasawa, H.; Suzuki, E.; Shimada, I. Collagen-binding mode of vwf-a3 domain determined by a transferred cross-saturation experiment. *Nat. Struct. Biol.* **2003**, *10*, 53–58.
43. Nummelin, H.; Merckel, M.C.; Leo, J.C.; Lankinen, H.; Skurnik, M.; Goldman, A. The yersinia adhesin yada collagen-binding domain structure is a novel left-handed parallel beta-roll. *EMBO J.* **2004**, *23*, 701–711.
44. Palma, P.N.; Lagoutte, B.; Krippahl, L.; Moura, J.J.G.; Guerlesquin, F. Synechocystis ferredoxin/ferredoxin-nadp(+)-reductase/nadp(+) complex: Structural model obtained by nmr-restrained docking. *FEBS Lett.* **2005**, *579*, 4585–4590.
45. Pauleta, S.R.; Cooper, A.; Nutley, M.; Errington, N.; Harding, S.; Guerlesquin, F.; Goodhew, C.F.; Moura, I.; Moura, J.J.G.; Pettigrew, G.W. A copper protein and a cytochrome bind at the same site on bacterial cytochrome c peroxidase. *Biochem.* **2004**, *43*, 14566–14576.
46. Pedroso, H.A.; Silveira, C.M.; Almeida, R.M.; Almeida, A.; Besson, S.; Moura, I.; Moura, J.J.; Almeida, M.G. Electron transfer and docking between cytochrome cd nitrite reductase and different redox partners—A comparative study. *Biochim. Biophys. Acta* **2016**, *1857*, 1412–1421.
47. Pettigrew, G.W.; Goodhew, C.F.; Cooper, A.; Nutley, M.; Jumel, K.; Harding, S.E. The electron transfer complexes of cytochrome c peroxidase from paracoccus denitrificans. *Biochemistry* **2003**, *42*, 2046–2055.
48. Pettigrew, G.W.; Pauleta, S.R.; Goodhew, C.F.; Cooper, A.; Nutley, M.; Jumel, K.; Harding, S.E.; Costa, C.; Krippahl, L.; Moura, I.; et al. Electron transfer complexes of cytochrome c peroxidase from paracoccus denitrificans containing more than one cytochrome. *Biochemistry* **2003**, *42*, 11968–11981.
49. Philominathan, S.T.L.; Koide, T.; Hamada, K.; Yasui, H.; Seifert, S.; Matsushita, O.; Sakon, J. Unidirectional binding of clostridial collagenase to triple helical substrates. *J. Biol. Chem.* **2009**, *284*, 10868–10876.
50. 50. Philominathan, S.T.; Koide, T.; Matsushita, O.; Sakon, J. Bacterial collagen-binding domain targets undertwisted regions of collagen. *Protein Sci.* **2012**, *21*, 1554–1565.

51. Pieulle, L.; Nouailler, M.; Morelli, X.; Cavazza, C.; Gallice, P.; Blanchet, S.; Bianco, P.; Guerlesquin, F.; Hatchikian, E.C. Multiple orientations in a physiological complex: The pyruvate-ferredoxin oxidoreductase-ferredoxin system. *Biochemistry* **2004**, *43*, 15480–15493.
52. Pieulle, L.; Morelli, X.; Gallice, P.; Lojou, E.; Barbier, P.; Czjzek, M.; Bianco, P.; Guerlesquin, F.; Hatchikian, E.C. The type i/type ii cytochrome c(3) complex: An electron transfer link in the hydrogen-sulfate reduction pathway. *J. Mol. Biol.* **2005**, *354*, 73–90.
53. Poletto, G.; Vilardell, J.; Marin, O.; Pagano, M.A.; Cozza, G.; Sarno, S.; Falques, A.; Itarte, E.; Pinna, L.A.; Meggio, F. Regulatory subunit of protein kinase ck2 contributes to the recognition of the substrate consensus sequence. A study with an eif2 beta-derived peptide. *Biochemistry* **2008**, *47*, 8317–8325.
54. Rajalingam, D.; Kumar, T.K.S.; Soldi, R.; Graziani, I.; Prudovsky, I.; Yu, C. Molecular mechanism of inhibition of nonclassical fgf-1 export. *Biochemistry* **2005**, *44*, 15472–15479.
55. Raychaudhuri, S.; Skommer, J.; Henty, K.; Birch, N.; Brittain, T. Neuroglobin protects nerve cells from apoptosis by inhibiting the intrinsic pathway of cell death. *Apoptosis* **2010**, *15*, 401–411.
56. Ribeiro Ede, A., Jr.; Leyrat, C.; Gerard, F.C.; Albertini, A.A.; Falk, C.; Ruigrok, R.W.; Jamin, M. Binding of rabies virus polymerase cofactor to recombinant circular nucleoprotein-rna complexes. *J. Mol. Biol.* **2009**, *394*, 558–575.
57. Rudolph, C.; Sigruener, A.; Hartmann, A.; Orso, E.; Bals-Pratsch, M.; Gronwald, W.; Seifert, B.; Kalbitzer, H.R.; Verdorfer, I.; Luetjens, C.M.; et al. Apoa-i-binding protein (ai-bp) and its homologues hyjef_n2 and hyjef_n3 comprise the yjef_n domain protein family in humans with a role in spermiogenesis and oogenesis. *Horm. Metab. Res.* **2007**, *39*, 322–335.
58. Saglietti, L.; Dequidt, C.; Kamieniarz, K.; Rousset, M.-C.; Valnegri, P.; Thoumine, O.; Beretta, F.; Fagni, L.; Choquet, D.; Sala, C.; et al. Extracellular interactions between glur2 and n-cadherin in spine regulation. *Neuron* **2007**, *54*, 461–477.
59. Skommer, J.; Brittain, T. Extended survival of sh-sy5y cells following overexpression of lys67glu neuroglobin is associated with stabilization of deltapsim. *Cytom. A* **2012**, *81*, 602–610.
60. Spies, M.; Kowalczykowski, S.C. The reca binding locus of recbcd is a general domain for recruitment of DNA strand exchange proteins. *Mol. Cell* **2006**, *21*, 573–580.
61. Srinivasan, M.; Lu, D.B.; Eri, R.; Brand, D.D.; Haque, A.; Blum, J.S. Cd80 binding polyproline helical peptide inhibits t cell activation. *J. Biol. Chem.* **2005**, *280*, 10149–10155.
62. Winkler, M.; Kuhlgert, S.; Hippler, M.; Happe, T. Characterization of the key step for light-driven hydrogen evolution in green algae. *J. Biol. Chem.* **2009**, *284*, 36620–36627.
63. Worrall, J.A.R.; Liu, Y.J.; Crowley, P.B.; Nocek, J.M.; Hoffman, B.M.; Ubbink, M. Myoglobin and cytochrome b(5): A nuclear magnetic resonance study of a highly dynamic protein complex. *Biochemistry* **2002**, *41*, 11721–11730.
64. Xu, X.; Schuermann, P.; Chung, J.-S.; Hass, M.A.S.; Kim, S.-K.; Hirasawa, M.; Tripathy, J.N.; Knaff, D.B.; Ubbink, M. Ternary protein complex of ferredoxin, ferredoxin:Thioredoxin reductase, and thioredoxin studied by paramagnetic nmr spectroscopy. *J. Am. Chem. Soc.* **2009**, *131*, 17576–17582.
65. Yabukarski, F.; Leyrat, C.; Martinez, N.; Communie, G.; Ivanov, I.; Ribeiro, E.A., Jr.; Buisson, M.; Gerard, F.C.; Bourhis, J.M.; Jensen, M.R.; et al. Ensemble structure of the highly flexible complex formed between vesicular stomatitis virus unassembled nucleoprotein and its phosphoprotein chaperone. *J. Mol. Biol.* **2016**, *428*, 2671–2694.
66. Zhu, S.Y.; Tytgat, J. Evolutionary epitopes of hsp90 and p23: Implications for their interaction. *FASEB J.* **2004**, *18*, 940–947.
